# The predictive capacity of polygenic risk scores for disease risk is only moderately influenced by imputation panels tailored to the target population

**DOI:** 10.1093/bioinformatics/btae036

**Published:** 2024-01-23

**Authors:** Hagai Levi, Ran Elkon, Ron Shamir

**Affiliations:** The Blavatnik School of Computer Science, Tel Aviv University, Tel Aviv 69978, Israel; Department of Human Molecular Genetics and Biochemistry, Sackler School of Medicine, Tel Aviv University, Tel Aviv 69978, Israel; Department of Human Molecular Genetics and Biochemistry, Sackler School of Medicine, Tel Aviv University, Tel Aviv 69978, Israel; Sagol School of Neuroscience, Tel Aviv University, Tel Aviv 69978, Israel; The Blavatnik School of Computer Science, Tel Aviv University, Tel Aviv 69978, Israel

## Abstract

**Motivation:**

Polygenic risk scores (PRSs) predict individuals’ genetic risk of developing complex diseases. They summarize the effect of many variants discovered in genome-wide association studies (GWASs). However, to date, large GWASs exist primarily for the European population and the quality of PRS prediction declines when applied to other ethnicities. Genetic profiling of individuals in the discovery set (on which the GWAS was performed) and target set (on which the PRS is applied) is typically done by SNP arrays that genotype a fraction of common SNPs. Therefore, a key step in GWAS analysis and PRS calculation is imputing untyped SNPs using a panel of fully sequenced individuals. The imputation results depend on the ethnic composition of the imputation panel. Imputing genotypes with a panel of individuals of the same ethnicity as the genotyped individuals typically improves imputation accuracy. However, there has been no systematic investigation into the influence of the ethnic composition of imputation panels on the accuracy of PRS predictions when applied to ethnic groups that differ from the population used in the GWAS.

**Results:**

We estimated the effect of imputation of the target set on prediction accuracy of PRS when the discovery and the target sets come from different ethnic groups. We analyzed binary phenotypes on ethnically distinct sets from the UK Biobank and other resources. We generated ethnically homogenous panels, imputed the target sets, and generated PRSs. Then, we assessed the prediction accuracy obtained from each imputation panel. Our analysis indicates that using an imputation panel matched to the ethnicity of the target population yields only a marginal improvement and only under specific conditions.

**Availability and implementation:**

The source code used for executing the analyses is this paper is available at https://github.com/Shamir-Lab/PRS-imputation-panels.

## 1 Introduction

Polygenic risk scores (PRSs) play a prominent role in the vision of precision medicine, offering significant potential for enhancing healthcare outcomes. They can predict individuals’ risk of developing a certain disease based on summary statistics of genome-wide association studies (GWASs). As ethnic groups differ in genetic structure, the set of individuals included in a GWAS, also called the discovery set, are usually from the same ethnicity. The PRS constructed from GWAS can then be used to calculate risk scores for another group of individuals, also called the target set. Ideally, the discovery and the target sets should come from the same ethnic group. The prediction accuracy of PRS decreases as the genetic distance between the discovery and the target set increases (Martin *et al.* 2019). At present, most GWASs have been compiled from the European (EUR) population (Martin *et al.* 2019), and applying the PRSs generated from them to non-EUR individuals has lower accuracy. Therefore, the health benefits that can be achieved using current PRS models are still not effectively applicable to most of the world’s population.

Genetic profiling of individuals in the discovery and target sets is usually obtained by SNP arrays that genotype a predefined subset of several hundreds of thousands of SNPs per individual. Nevertheless, additional information is often contained in millions of other SNPs that were not genotyped. Furthermore, discovery and target sets are often genotyped by different arrays. Computationally calling non-genotyped SNPs is done using imputation, a family of methods that infer untyped SNPs using a set of fully sequenced individuals for whom several millions of SNPs are called (Yun *et al.* 2009, Chen *et al.* 2020). That set of individuals is called the imputation panel. Imputation methods rely on the fact that chromosome recombination during evolution has generated genomic intervals within the human genome where proximal SNPs tend to be inherited together, a phenomenon called linkage disequilibrium (LD). Imputation methods use the panel to complete untyped SNPs by assuming common LD structure between the individuals in the target (genotyped) and the panel (fully sequenced) sets (Howie *et al.* 2009).

Several studies investigated properties that affect the accuracy of the imputation process, including genotyping platform (Nelson *et al.* 2013, Hanks *et al.* 2022), imputation panels (Hanks *et al.* 2022), imputation methods (Stahl *et al.* 2021) and imputation servers (Sengupta *et al.* 2023). The panels tested in these studies typically consist of predominantly EUR individuals [e.g. HRC (McCarthy *et al.* 2016)] or EUR mixed with other populations [e.g. 1000 Genomes (Auton *et al.* 2015), HapMap (Rotimi *et al.* 2007), and TOPMed (Taliun *et al.* 2021)]. Therefore, properties of the genetic structure [e.g. LD structure and minor allele frequency (MAF)] might differ between these panels and non-EUR populations. Indeed, such panels typically yield lower imputation accuracy on non-EUR population (Cahoon *et al.* 2023). Other studies showed that using an ethnic-matched imputation panel improves the accuracy of completing untyped SNPs in specific populations, including Ashkenazi Jews (Lencz *et al.* 2018), Africans (Sengupta *et al.* 2023), East Asians (Bai *et al.* 2020), and South Asians (Ahmad *et al.* 2017). Nevertheless, accurately completing untyped SNPs does not necessarily imply better risk prediction with PRS. Moreover, rare SNPs (<1% MAF) are more sensitive to the choice of the imputation process (Lencz *et al.* 2018, Shi *et al.* 2018), but PRSs rely mainly on common SNPs. While some works examined factors in the imputation process that impact the quality of PRS, such as the genotyping platform, phasing, and imputation methods (Chen *et al.* 2020, Thanh Nguyen *et al.* 2022), there has been no systematic assessment of how different ethnic compositions of the imputation panel affect the accuracy of PRS risk prediction.

In this study, we estimated the effect of the ethnicities of the imputation panel and the target set on the prediction accuracy of PRS in cases where the discovery and the target sets come from different ethnic groups. We analyzed 12 binary phenotypes and three populations from the UK Biobank (UKB) (Europeans, South-Asian, and Africans). We generated imputation panels from several ethnic groups, imputed the target set according to each panel, used PRS to compute individuals’ risk scores, and compared the performance of risk prediction using each panel.

## 2 Results

We started by evaluating the quality of imputation as a function of the ethnic composition of the imputation panel and the imputed individuals. For a given imputation panel and a test set of fully sequenced individuals, we masked all SNPs in the test set except those included in the UKB genotyping chip (*n* = 784 849) (Bahcall 2018). Then, we imputed the masked SNPs using the imputation panel, and computed the fraction of the SNPs that were correctly imputed. We call that fraction the imputation accuracy.

To generate imputation panels for different ethnicities, we used the 1000 Genomes Project dataset (Auton *et al.* 2015), which contains sequences of ∼2500 individuals annotated by their country (subpopulation) and continent (super-population) of origin. For each super-population, we took five groups of 70 individuals from each of its five subpopulations to generate an imputation panel (*n* = 350). Using these sets, we generated an imputation panel for each super-population: EUR, East Asians (EAS), Africans (AFR), and South Asians (SAS).

Similarly, for each super-population, we generated a test set by collecting 100 individuals from it, 20 from each subpopulation (Supplementary Table S1). In addition, we generated another target set of two African subpopulations from the 1000 Genomes that do not appear in the African imputation panel (*n* = 145; AFR2; Supplementary Table S1). The target sets and the imputation panels were disjoint. After masking SNPs that do not appear on the UKB chip, we imputed each target set using the different imputation panels we generated, and calculated the imputation accuracy. This test was conducted for each autosomal chromosome separately.

**Figure 1.**
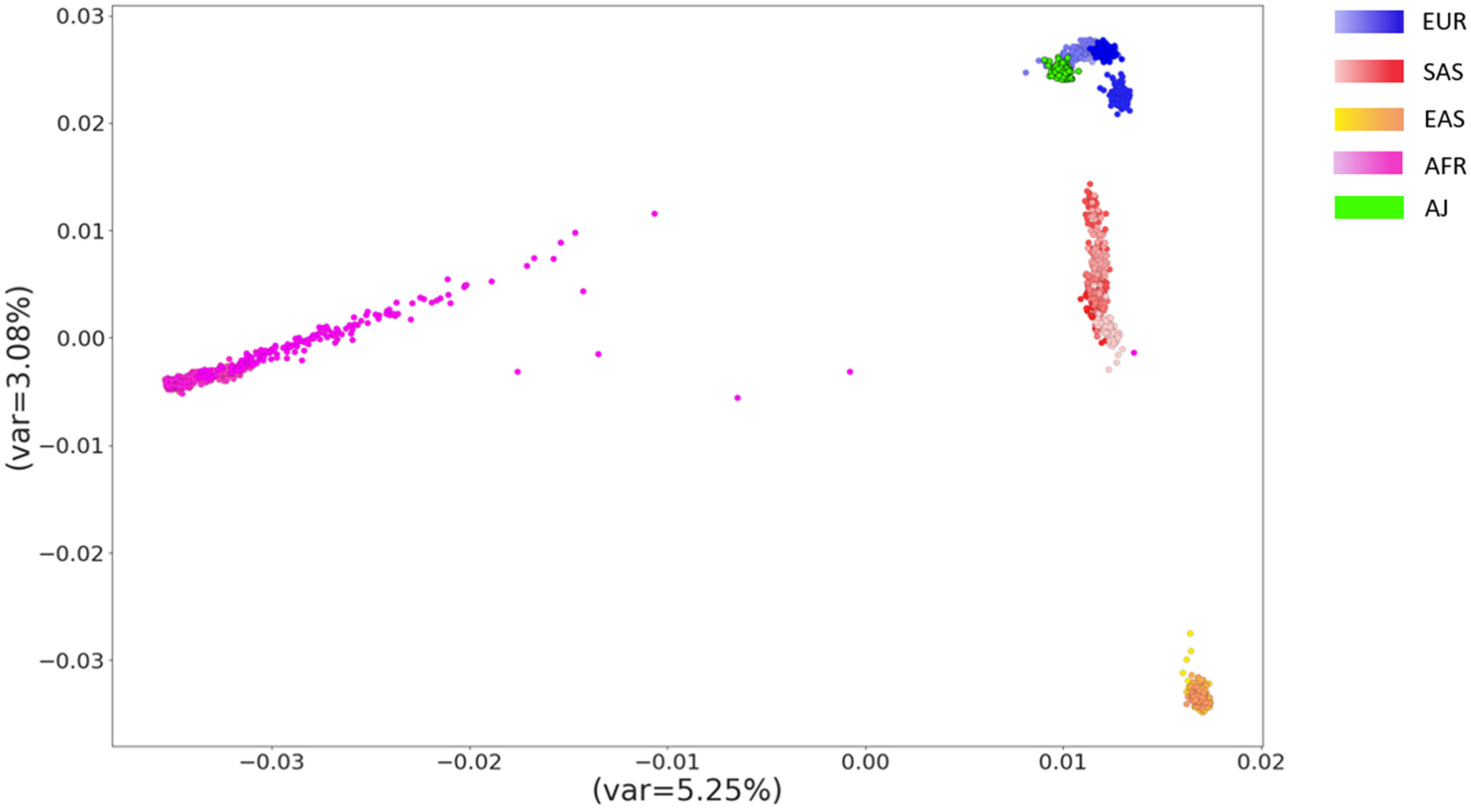
Principal component analysis on genotypes of four super-populations from the 1000 Genomes project (*n* = 2157, Auton *et al.* 2015) and Ashkenazi Jews (*n* = 128, Carmi *et al.* 2014), demonstrating genetic distances between populations (EUR, European; EAS, East Asians; SAS, South Asians; AFR, Africans; AJ, Ashkenazi Jews)

Table 1 shows the average imputation accuracy on different populations when using imputation panels of different ethnicities. The imputation accuracy deteriorates as the genetic distance $[F_{st}$; calculated using (Bhatia *et al.* 2013); Table 2] between the ethnic groups increases. The genetic distance can also be visualized using principal component analysis (PCA) of the SNPs (Fig. 1). For example, the closest super-population to EUR is SAS ($F_{st}$ = 0.03), then EAS (0.1), and the farthest is AFR (0.12). Accordingly, imputation accuracy obtained on the EUR test set was 0.99, 0.982, 0.957, and 0.947 using EUR, SAS, EAS, and AFR imputation panels, respectively. The agreement between distance and imputation accuracy also holds for other super-populations and on each chromosome individually (Supplementary Table S2).

**Table 1.** Accuracy of imputing different populations using panels from different ethnicities.^a^

	EUR	EAS	SAS	AFR	AFR2
**EUR**	**0.989**	0.960	0.969	0.817	0.822
**EAS**	0.955	**0.987**	0.960	0.808	0.813
**SAS**	0.981	0.971	**0.984**	0.809	0.815
**AFR**	0.946	0.920	0.926	**0.940**	**0.930**

a Columns: The imputed population. Rows: The ethnicity of the imputation panel. The numbers are the accuracy averaged across chromosomes (best performing panel for each test set is in bold).

**Table 2.** Genetic distance (Hudson’s $F_{ST}$) between ethnic groups.

	AFR	SAS	EAS	EUR	AJ
**AFR**		0.12	0.15	0.12	0.12
**SAS**			0.06	0.03	0.04
**EAS**				0.1	0.11
**EUR**					0.01
**AJ**					

Two additional observations emerged from this analysis: (i) The highest accuracy in African individuals—obtained using the AFR imputation panel—was substantially lower than the accuracy in other ethnic groups. For example, the accuracy obtained in the AFR and AFR2 sets was 0.94 and 0.93, respectively, whereas for the EUR, EAS, and SAS sets, when using ethnically matched imputation panels, the accuracy was 0.989, 0.987, and 0.984, respectively. (ii) An AFR imputation panel can be used for imputing EUR genotypes with a limited decrease in accuracy (0.989 versus 0.946). However, the decrease in the other direction is much larger: using a non-AFR panel for imputing AFR individuals causes a large drop in the imputation accuracy (e.g. 0.94 using the AFR panel versus 0.817 using the EUR panel). Similar results have been reported in previous studies (Zhang *et al.* 2011). These findings reflect the more complex genetic structure of the AFR population compared to non-AFR populations.

Next, to include in our analyses a population that is more closely related to EUR, we added a cohort of fully sequenced Ashkenazi Jewish (AJ) individuals from (Carmi *et al.* 2014), and conducted a similar analysis on this ethnic group. We generated an AJ imputation panel from 100 individuals and tested the imputation accuracy on a disjoint set of 27 individuals from the same study. In addition, we generated three imputation panels, each comprising 100 individuals from each of the EUR, EAS, and AFR super-populations from the 1000 Genomes and used them to impute the same 27 AJ genotypes. Generally, the results of this analysis (Table 3, Supplementary Table S3) were consistent with those presented above. The only exception was that the AFR imputation panel did slightly better than the EAS imputation panel. Notably, both populations are very far from the AJ ($F_{st}=0.11$ and 0.12 for EAS and AFR, respectively).

**Table 3.** Imputation accuracy on an Ashkenazi Jewish (AJ) test set using panels from different ethnicities, and the genetic distance (Hudson’s $F_{ST}$) between AJ to the other ethnic groups.

	Imputation accuracy	Genetic distance
**AJ**	0.956	—
**EUR**	0.951	0.01
**EAS**	0.917	0.11
**AFR**	0.928	0.12

**Figure 2.**
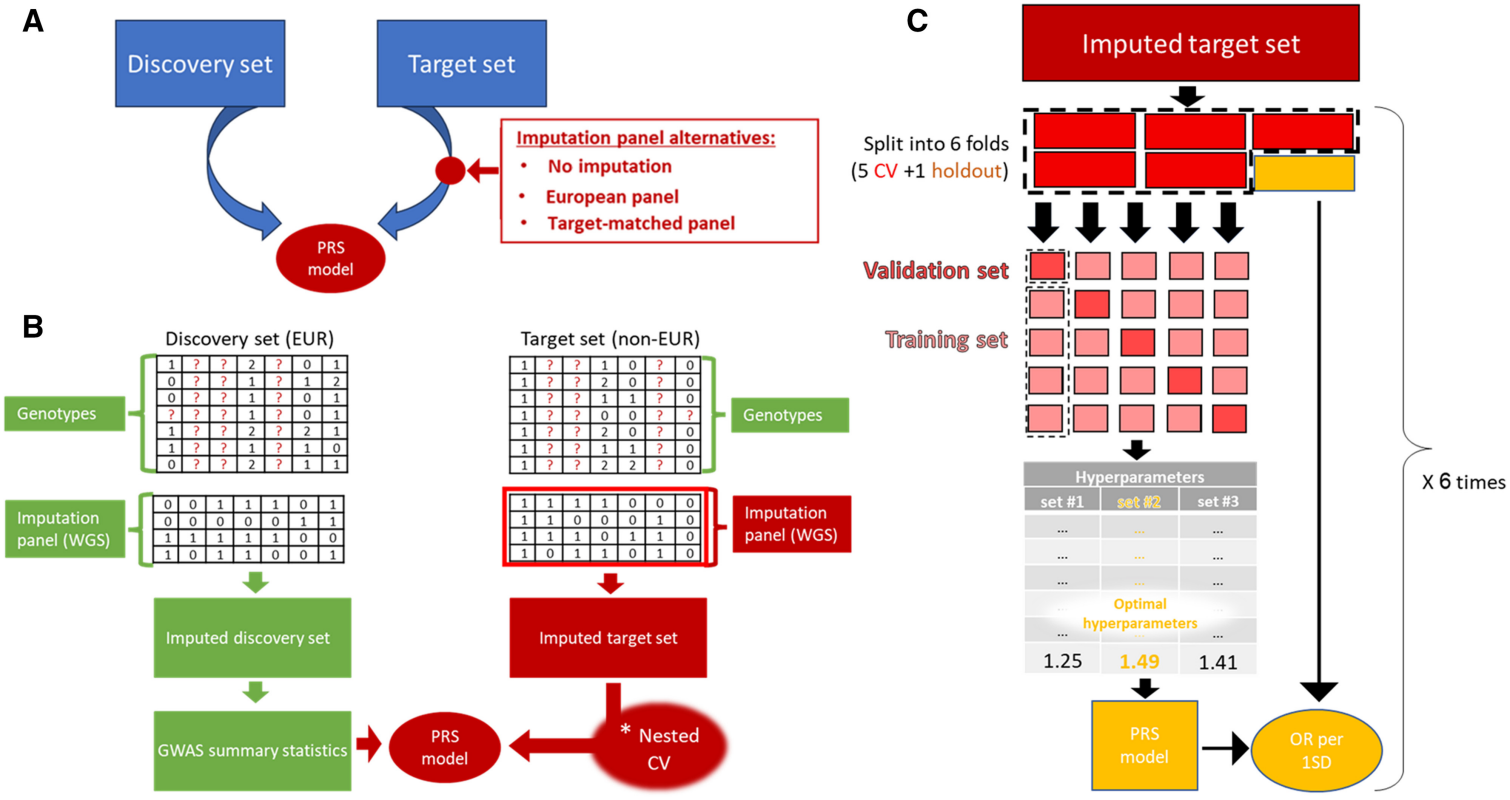
The procedure for evaluating the effect of the imputation panel on PRS prediction performance. (**A**) High-level of the PRS generation process. The red box shows the possible options of the imputation panel. (**B**) Detailed description of our PRS pipeline. Parts in green are fixed throughout the analysis, while those in red are affected by the choice of the imputation panel. (**C**) The nested cross-validation (CV) process. Briefly, the imputed target set is split randomly into six equal sized sets. One set is held out (yellow; outside the thick dashed outline) and the other five (red; inside the thick dashed outline) are used to perform a standard 5-fold CV, in which four out of five parts are used to derive PRS models with different predefined sets of hyper-parameters (light red; small squares), and then the resulting models are applied on the fifth part. After iterating over the five combinations of training and test sets, the best performing hyper-parameter set is chosen. The resulting PRS model is used on the held-out set. The entire process is repeated six times, with the different held-out sets, and the average performance is computed (yellow oval).

The analyses above suggest that imputing genotypes using an ethnic-matched panel is more accurate. Next, we sought to examine the effect of the imputation panel on the performance of PRS prediction when applied to target sets of different ethnicities than the discovery (GWAS) population. The outline of the evaluation pipeline is depicted in Fig. 2. We used UKB data and focused on EUR (*n* = 472 694) as a discovery set, with SAS and AFR as target sets (*n* = 9881, *n* = 8060, respectively). Twelve diseases that had sufficient representation of SAS and AFR cases were included in our analysis (Supplementary Table S4; Section 4). For each disease, we generated GWAS from the discovery set, created a PRS and tested its prediction quality on the target sets imputed using three imputation panels. Here and throughout the rest of our study, each imputation panel was generated using all the available samples from the relevant super-population in the 1000 Genomes project (Fig. 2A; Section 4).

We applied four methods for PRS construction—P + T with discovery-set LD, P + T with target-set LD, Lassosum, and LDpred2 (Section 4)—and evaluated PRS performance using a nested cross-validation scheme (Fig. 2B and C). Since LDpred2 is computationally intensive, we executed it with a lighter version of our pipeline (Section 4). We calculated the odds ratio per 1 unit of standard deviation (OR per 1SD) to measure the quality of the PRS prediction. We also evaluated the performance of PRSs that were built only on SNPs directly typed by the SNP array (i.e. without imputed SNPs). Lassosum and LDpred2 obtained the highest results, although some of LDpred2 executions did not terminate successfully (Section 4). Therefore, below we mainly focus on results obtained using Lassosum.

**Figure 3.**
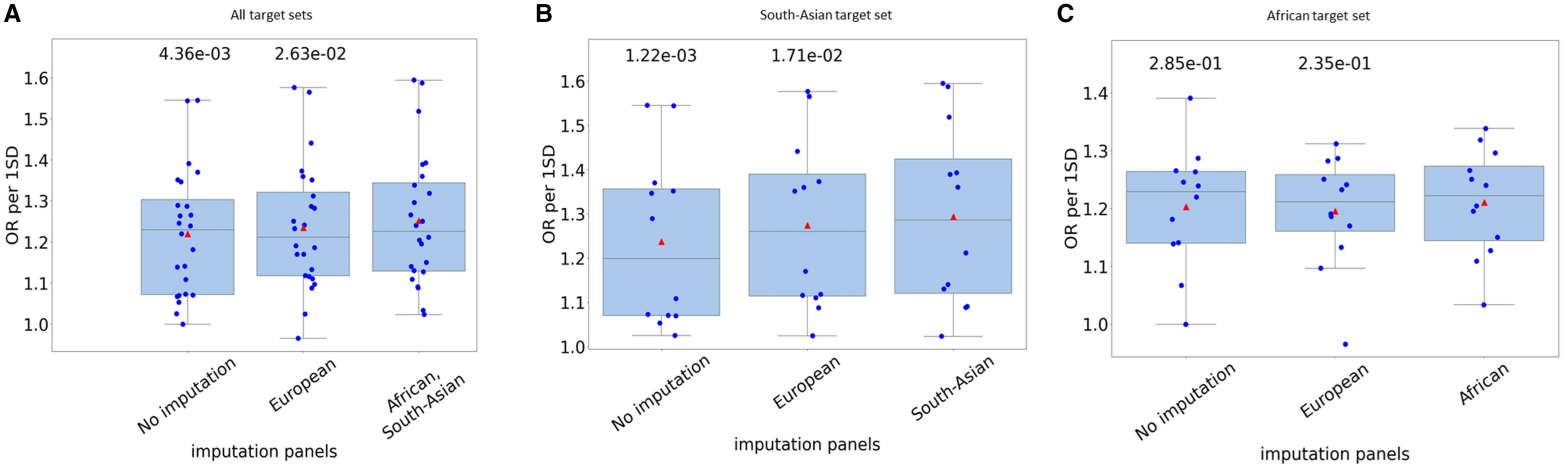
The effect of ethnic composition of the imputation panel on the performance of PRS constructed by the Lassosum method when applied to a target set of a different ethnicity than the GWAS. The graphs show OR per 1SD of 12 traits. PRSs were built from GWASs computed on UKB EUR individuals. Results are shown for (**A**) both SAS and AFR (**B**) SAS only (**C**) AFR only. The *P*-values above each boxplot compare the results with the imputation panel listed below it to the results with the imputation panel that matches the population of the target set. *P*-values were calculated using one-tailed Wilcoxon test. Triangles are the averages.

In the first test, we imputed each individual from the SAS and AFR populations with a target-matched imputation panel, that is, SAS (AFR) individuals were imputed using the SAS (AFR) imputation panel. Overall, significantly higher OR per 1SD values were obtained with Lassosum in target-matched imputed sets compared to the EUR imputed set and the non-imputed set (Fig. 3A). Next, we looked at the same results when the individuals are split into separate target sets according to their ethnicity. Here too, a higher average OR per 1SD was observed on both SAS and AFR target sets, but only the effect observed for the SAS population reached statistical significance (Fig. 3B and C). Most of the results obtained using other PRS methods did not consistently show improvement for the target-matched imputation panel (Supplementary Figs S1 and S2).

To reaffirm these results, we used publicly available EUR-predominant GWASs that were not constructed using the UKB data to generate PRS models. We chose five traits that had a sufficient number of cases in the UKB SAS and AFR cohorts (see Section 4). Using the same pipeline as above, we generated PRS models from these public GWASs and calculated their performance on the SAS and AFR target sets from the UKB, imputed with different ethnic panels. The results are summarized in Table 4. For SAS, only one PRS method obtained higher OR per 1SD using the target-matched imputation panel than those obtained using the EUR imputation panel. Surprisingly, for AFR target sets, the PRSs calculated using P + T (with both LD options) were the highest in non-imputed genotypes. Overall, the differences between the imputation panel alternatives were marginal. As in our previous analysis, the results for AFR population are more complex.

**Table 4.** Quality of PRS models on the UKB SAS and AFR cohorts, computed using different imputation panels and PRS methods.^a^

Target set	PRS method	Imputation panel	OR per 1SD
**South-Asian**	Lassosum	European	1.41 ± 0.272
		No imputation	1.38 ± 0.258
		South-Asian	1.41 ± 0.275
	P + T (target set LD)	European	1.38 ± 0.219
		No imputation	1.39 ± 0.265
		South-Asian	1.4 ± 0.283
	P + T (EUR LD)	European	1.31 ± 0.164
		No imputation	1.27 ± 0.145
		South-Asian	1.31 ± 0.149
**African**	Lassosum	African	1.27 ± 0.15
		European	1.25 ± 0.132
		No imputation	1.26 ± 0.117
	P + T (target set LD)	African	1.25 ± 0.192
		European	1.2 ± 0.121
		No imputation	1.25 ± 0.177
	P + T (EUR LD)	African	1.17 ± 0.137
		European	1.17 ± 0.1
		No imputation	1.18 ± 0.097

a PRSs were built using EUR-predominant GWASs (*n* = 5) that were not computed with UKB data. P + T: Pruning and thresholding. For P + T, we tested the performance using both EUR LD and the target set LD.

Next, we examined the performance of PRS for Schizophrenia (SCZ), built from EUR SCZ GWAS, in two non-UKB target sets: Ashkenazi Jews and Africans (see Section 4; Supplementary Table S5). We imputed these target sets using three imputation panels from different ethnic groups: EUR, EAS, and AFR. Here too, we ran the nested cross-validation scheme to calculate OR per 1SD for each PRS model (see Section 4). The results are shown in Table 5. In five out of six cases, using target-matched imputation panels yielded the highest or second best PRS performance, with a marginal gap of ≤ 0.02. Here too, the differences in PRS performance were not statistically significant.

**Table 5.** OR per 1SD of European Schizophrenia PRS methods with three imputation panels on non-UKB target sets.^a^

Imputation panel	Method	AFR target set	AJ SCZ target set
AFR	P + T (EUR LD)	1.28 ± 0.048	2.09 ± 0.06
EAS		1.46 ± 0.108	2.06 ± 0.086
EUR		1.5 ± 0.114	2.07 ± 0.074
AFR	P + T (Target LD)	1.6 ± 0.143	2.11 ± 0.088
EAS		1.54 ± 0.129	2.05 ± 0.06
EUR		1.34 ± 0.097	2.2 ± 0.093
AFR	Lassosum	1.6 ± 0.126	2.51 ± 0.086
EAS		1.50 ± 0.163	2.47 ± 0.089
EUR		1.46 ± 0.119	2.50 ± 0.072

a Note that for AJ, the EUR imputation panel is the one considered target-matched.

Next, we tested the effect of imputation panel using non-EUR PRSs. We ran our evaluation pipeline with nine EAS GWASs from (Sakaue *et al.* 2021), each matching one of the 12 traits we investigated in the UKB target sets (Supplementary Fig. S3, Supplementary Table S4). In addition, we performed a similar test with EAS SCZ GWAS (Lam *et al.* 2019) on the two non-UKB SCZ target sets described above (AJ and AFR) (Supplementary Table S6). In all the analyses made using EAS GWASs, we could not identify an imputation panel that systematically yields better PRS performance. Notably, the PRS performance obtained with EAS-GWAS were generally lower compared to those of the UKB GWASs. This is probably due to the larger genetic distance between the discovery and the target sets (Table 2), and to the smaller EAS discovery set. This supports our expectation that the potential advantage of the target-matched imputation panel is undermined when the PRS performance is poor.

**Figure 4.**
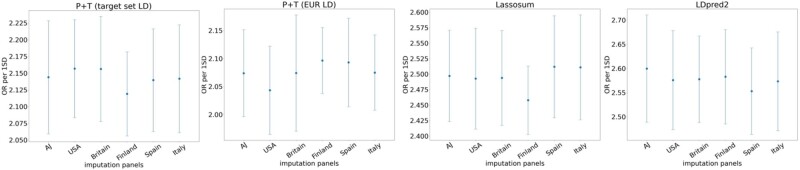
Performance of the EUR-SCZ PRS on the AJ target set imputed using imputation panels derived from different EUR subpopulations. OR per 1SD was computed by several PRS methods using different imputation panels. Dots represent the average value obtained using nested CV, and error bars indicate ±1 SEM

Last, we explored the effect of imputation panels that contain homogenous subpopulations. We imputed the SCZ AJ target set from (Lencz *et al.* 2013) using five imputation panels, each compiled from a different EUR subpopulation from the 1000 Genomes project (Italy, Spain, Finland, UK, and the US), and another imputation panel generated from 100 AJ individuals. As before, ORs per 1SD were calculated according to our nested cross-validation scheme. Here too, PRS performance with different imputation panels was comparable (Fig. 4), likely since the genetic distance between the subpopulations is low.

## 3 Discussion

In the emerging era of precision medicine, health disparity has become a major concern. As most available genotypes are predominantly from European population, clinically relevant PRS models are available mainly for that population. Often, many SNPs pertinent to the PRS are missing from the target genotypes. Hence, the imputation process, in which untyped SNPs are completed, could potentially affect the PRS prediction performance.

In this study, we first reconfirmed that imputing genotypes using an ethnic-matched panel is more accurate. However, more accurate SNP imputation does not necessarily imply better risk prediction with PRS. PRS performance deteriorates as the genetic distance between the discovery and the target sets increases (Martin *et al.* 2019). Therefore, the optimal imputation panel for accurately completing missing SNPs may not necessarily give superior PRS performance when the discovery and the target sets come from different populations.

When utilizing European GWASs derived from UKB data, we observed improved PRS performance primarily when the target set was imputed using a panel from the same ethnic background. This general trend reached statistical significance when tested using Lassosum on AFR and SAS target sets combined, as well as on the SAS target set separately (Fig. 3).

While matching the imputation panel to the target population tended to boost PRS performance, the improvement was limited. The statistically significant increase in the OR per 1SD in Lassosum was less than 0.1 on average, suggesting that even when a target-matched imputation panel significantly improves the PRS performance, the magnitude of this effect is moderate. Moreover, when P + T PRS methods were tested, the effect vanished (Supplementary Fig. S1). Notably, the P + T methods were consistently inferior to Lassosum, suggesting that the benefit of using a target-matched imputation panel is apparent when using more advanced PRS methods.

The improvement obtained by using target-matched imputation panel was more noticeable on the SAS population than the AFR one. We suspect that the substantial genetic distance between the EUR population, from which the GWAS was constructed, and the AFR population is the reason why accurately completing missing SNPs did not lead to a significant improvement in EUR-based PRS performance on AFR population.

Nevertheless, the superiority of target-matched imputation panel diminished in EUR-predominant non-UKB GWASs (Table 4) and non-UKB target sets (Table 5), and dissipated completely in EAS GWASs (Supplementary Fig. S3, Supplementary Table S6) and subpopulation-specific imputation panels (Fig. 4). These results suggest that the potential of target-matched imputation panel to improve PRS performance might be hindered by many factors, such as the genetic distance between the discovery and the target sets, different genotyping platforms, low-powered GWAS, and modest genetic variation among the alternative imputation panels. As was previously described (Zhang *et al.* 2011, Shi *et al.* 2018), increasing the size of the imputation panel beyond 100–200 also did not lead to improved PRS performance (Supplementary Fig. S4).

The UKB contains approximately 8000 and 10 000 AFR and SAS participants, respectively. We analyzed only 12 diseases that had at least 200 cases in either the AFR or SAS cohorts. This limited our ability to reach statistical significance in that analysis. Future studies can focus on continuous traits and thus alleviate this problem.

To date, reliable PRSs have been developed mostly for EUR individuals. A major effort is currently made to conduct GWASs for non-EUR populations in order to produce clinically relevant PRSs for those ethnicities. This requires collecting large cohorts of cases and controls for each phenotype and each non-EUR population, which is expensive, logistically complex, and sometimes not feasible for small minorities. Our findings point out that when the target population is moderately close to the EUR population, using an ethnically matched imputation panel has the potential to enhance the prediction performance of European-based PRSs.

## 4 Materials and Methods

### 4.1 Target sets

We generated two non-EUR super-population target sets from the UKB: For Africans and South Asians. Twelve traits were tested on those target sets (Supplementary Table S4). In addition, we used two non-UKB SCZ target sets obtained from dbGaP: (i) SCZ Ashkenazi Jews from (Lencz *et al.* 2013). (ii) SCZ Africans from GAIN (Shi *et al.* 2009) (Supplementary Table S5).

### 4.2 Building imputation panels for UKB and GAIN genotypes

We used SHAPEIT2 (Delaneau *et al.* 2011) to generate imputation panels for four super-populations: EUR (*n* = 503), SAS (*n* = 489), EAS (*n* = 504), and AFR (*n* = 661). Each panel comprised all the individuals from its population in the 1000 Genomes project.

### 4.3 Building imputation panels for analyses involving AJ genotypes

To build an imputation panel for AJ, we used whole genome sequencing (WGS) data of 100 AJ individuals from (Carmi *et al.* 2014). In analyses that compared the performance of AJ imputation panel to other panels (Fig. 4, Table 5, Supplementary Table S6), we generated imputation panels comprising only SNPs that were found in both the AJ dataset and 1000 Genomes. To do so, we used BCFTOOLS (Danecek *et al.* 2021) to annotate the SNP in the AJ dataset with SNP ids (rsid) from the 1000 Genomes dataset. In this process, 8 637 756 SNPs in the AJ dataset were assigned with rsid. In all imputation panels used for imputing the AJ target set, we kept only SNPs were shared between the AJ and 1000 Genomes datasets. Then, we used SHAPEIT2 (Delaneau *et al.* 2011) to phase the remaining SNPs in the AJ dataset. Finally, we used SHAPEIT2 to generate imputation panels.

### 4.4 Genotype imputation

Using the imputation panels we generated (see above), we imputed genotypes from the UKB, GAIN (O’Donovan *et al.* 2008), and the SCZ-AJ datasets (Lencz *et al.* 2013). Imputation was done using IMPUTE2 (Howie *et al.* 2011).

### 4.5 Nested CV scheme

We applied a variant of the nested 6X5 CV scheme described in (Levi *et al.* 2023). Briefly, we split each target set cohort into six sets. Next, we held out one set and used the other five sets to perform a standard 5-fold CV, in which four out of five parts are used to derive PRS models with different predefined sets of hyper-parameters, and then the resulting models are applied on the fifth part. After iterating over the five combinations of training and test sets, we chose the best performing hyper-parameter set (see below). Finally, we applied the resulting PRS model on the held-out set. We repeated this entire process six times, each with a different held-out set and took the average. Due to its computational demands, LDpred2 was assessed in the UKB analysis using a 3 × 2 nested cross-validation.

### 4.6 Criteria for choosing an optimal PRS model

For each PRS method, we tested the performance with a predefined set of hyper-parameters (see below). We used the same criteria as in (Levi *et al.* 2023). Briefly, we ranked runs with different hyper-parameters using two metrics: (i) OR per 1SD. Here, the scores were standardized w.r.t. the controls in the target set. (ii) top-10% OR relative to the middle quintile. We combined these rankings by taking their sum and broke ties using OR per 1SD, our main metric.

### 4.7 PRS methods

We used four PRS methods: (i) P + T (EUR LD): Using PLINK, we clumped the GWAS results according to LD in the EUR population, and then we filtered the remaining SNPs based on a significance threshold. (ii) P + T (Target LD): Here, when applying LD clumping in PLINK, we used LD inferred from the training set. The rest is similar to the previous method. (iii) Lassosum: we generated a PRS model using a reference panel calculated from genotype data (i.e. the training set). (iv) LDPred2 (grid mode). We supplied LDpred2 with a training set that comes from the same population as the target set. As LDPred2 is computationally intensive, we executed LDpred2 only the evaluation in EUR UKB (Supplementary Fig. S2) and the subpopulation imputation panel analysis (Fig. 4). The EUR UKB evaluation was executed with a less computationally intensive version of our pipeline (see above). Since not all LDpred2 executions completed successfully, we included only traits for which we were able to infer LDpreds2 performance in the three imputation panel alternatives [SAS (*n* = 6); Supplementary Table S4].

### 4.8 Generating GWAS summary statistics from the UKB

We kept only SNPs with MAF ≥ 1%, HWE *P*-value ≤ 1e−6 and missing rate ≤10%. In addition, we kept only samples where less than 10% of SNPs present in the set were missing. We filtered out ambiguous and duplicated alleles. We built EUR GWAS for each binary phenotype encoded in data-field 20002 (non-cancer illness code). To guarantee the GWASs are well-powered, we considered only phenotypes with >7500 cases (Supplementary Table S3). In addition, to avoid high error estimates in our performance evaluations, we filtered out GWASs where the number of cases did not exceed 200 in neither AFR nor SAS target sets (Supplementary Table S3). A total of 12 phenotypes met these conditions. For each phenotype, we generated GWAS summary statistics by applying PLINK’s—assoc command to the imputed genotypes of the EUR population provided by the UKB.

### 4.9 GWAS summary statistics from public datasets

For the analysis of non-UKB EUR GWAS (Table 4), we used publicly available EUR-predominant GWAS for five traits that have at least 200 AFR and SAS cases in UKB: systolic blood pressure (BP) (Evangelou *et al.* 2018), cholesterol levels (LDL) (Willer *et al.* 2013), type 2 diabetes (Mahajan *et al.* 2018), gastric reflux (An *et al.* 2019), and major depression (Howard *et al.* 2019). For the analysis of SCZ PRS on non-UKB target sets (Table 5, Fig. 3), we used a leave-one-out version of the SCZ GWAS from (Ripke *et al.* 2014), where individuals presented in the AJ target set were excluded. For every public GWAS, we kept only SNPs with MAF ≥ 1%.

## Supplementary Material

btae036_Supplementary_Data

## Data Availability

The 1000 Genomes data used in this article are available in https://www.internationalgenome.org/. The EAS GWASs were derived from the PGS catalog: https://www.pgscatalog.org/. The AFR and AJ SCZ genotypes were derived by permission from dbgap (https://www.ncbi.nlm.nih.gov/gap/) using access ids phs000021.v3 phs000448.v1.p1, respectively. The AJ WGS data used for generating AJ imputation panel were provided by permission by The Ashkenazi Genome Consortium: https://www.ebi.ac.uk/ega/datasets/EGAD00001000781. The UK biobank data were used by permission from https://www.ukbiobank.ac.uk/.
