# Supplementary material for: The predictive capacity of polygenic risk scores for disease risk is only moderately influenced by imputation panels tailored to the target population
Source: Bioinformatics. 2024 Jan 23;40(2):btae036. doi: 10.1093/bioinformatics/btae036 (PMC10868313; doi:10.1093/bioinformatics/btae036)
Supplement: btae036_Supplementary_Data [file btae036_supplementary_data.zip › supplementary.docx]

**The predictive capacity of polygenic risk scores for disease risk is only moderately influenced by imputation panels tailored to the target population**

Hagai Levi^1,2^, Ran Elkon*^,2^, Ron Shamir*^,1^

1. Blavatnik School of Computer Science, Tel Aviv University, Tel Aviv, Israel.

2. Department of Human Molecular Genetics and Biochemistry, Sackler School of Medicine, Tel Aviv University, Tel Aviv, Israel.

* Equal contribution

**Supplementary Information**

**Table S1. Ethnic groups from the 1000 Genomes that comprise the super-populations in the analyses described in Tables 1-3, S2-3.**

| **EUR** | **SAS** | **EAS** | **AFR** | **AFR2** |
| --- | --- | --- | --- | --- |
| GBR (British from England and Scotland) | BEB (Bengali in Bangladesh) | CDX (Chinese Dai in Xishuangbanna, China) | ACB (African Caribbean in Barbados) | MSL (Mende in Sierra Leone) |
| FIN (Finnish in Finland) | GIH (Gujarati Indians in Houston, Texas, USA) | CHB (Han Chinese in Beijing, China) | ESN (Esan in Nigeria) | ASW (African Ancestry in SW USA) |
| TSI (Toscani in Italia) | ITU (Indian Telugu in the U.K.) | CHS (Han Chinese South) | GWD (Gambian in Western Division – Mandinka) |  |
| IBS (Iberian populations in Spain) | PJL (Punjabi in Lahore, Pakistan) | JPT (Japanese in Tokyo, Japan) | LWK (Luhya in Webuye, Kenya) |  |
| CEU (Utah residents (CEPH) with northern and western European ancestry) | STU (Sri Lankan Tamil in the UK) | KHV (Kinh in Ho Chi Minh City, Vietnam) | YRI (Yoruba in Ibadan, Nigeria) |  |

**Table S2. See the file Table S2.xlsx**

**Table S3. See the file Table S3.xlsx**

**Table S4. Statistics for the traits tested on the UKB. All traits are binary. Cases were determined by the UKB records (field 20002). GWASs were constructed using EUR samples (n=****472,694), and target sets were generated for the AFR (n=8,060) and SAS populations (n=9,881) in the UKB.**

| **Trait** | **Number of EUR cases used for generating GWASs** | **Number of AFR cases** | **Number of SAS cases** |
| --- | --- | --- | --- |
| Angina* | 15242 | 197 | 516 |
| Asthma*^†^ | 54323 | 917 | 1134 |
| High cholesterol*^†^ | 61800 | 843 | 1817 |
| Cataract* | 9594 | 221 | 338 |
| gastro-esophageal reflux (GERD) / gastric reflux* | 22782 | 230 | 423 |
| Hay fever*^†^ | 29309 | 470 | 531 |
| Hypertension^†^ | 123613 | 2828 | 2763 |
| Hypothyroidism/Myxedema* | 23584 | 167 | 556 |
| Major depression disorder* | 29106 | 236 | 379 |
| Osteoarthritis^†^ | 41850 | 381 | 499 |
| Type 2 Diabetes*^†^ | 19129 | 753 | 1451 |
| Urinary tract infection | 7636 | 446 | 115 |

* Matching EAS GWAS were found for the trait

^†^ Results were obtained using LDpred2

**Table S5. Description of non-UKB SCZ target sets.**

| **Population** | **dbGaP access id** | **Number of cases** | **Number of controls** | **Genotyping platform** | **# of SNPs genotyped** |
| --- | --- | --- | --- | --- | --- |
| Ashkenazi Jews | phs000448.v1.p1 | 1044 | 2052 | Illumina’s HumanOmni1-Quad_v1-0_B | 1051295 |
| African | phs000021.v3 | 921 | 954 | Illumina’s AFFY_6.0 | 934940 |

**Table S6.** OR per 1SD for two non-UKB target sets when PRSs were generated from EAS SCZ GWAS. Lassosum was tested with ASN LDBlocks (hg19)

| Imputation panel | Method | AFR | AJ |
| --- | --- | --- | --- |
| AFR | P+T (EAS LD) | 1.07±0.022 | 0.95±0.026 |
| EAS |  | 0.941±0.011 | 0.95±0.028 |
| EUR |  | 1.002±0.025 | 0.922±0.053 |
| AFR | P+T (Target LD) | 1.019±0.039 | 1.02±0.034 |
| EAS |  | 1.041±0.051 | 1.01±0.027 |
| EUR |  | 1.075±0.058 | 1.04±0.042 |
| AFR | Lassosum (ASN) | 1.01±0.02 | 1.04±0.049 |
| EAS |  | 0.99±0.024 | 1.12±0.048 |
| EUR |  | 1.08±0.069 | 1.07±0.044 |

**
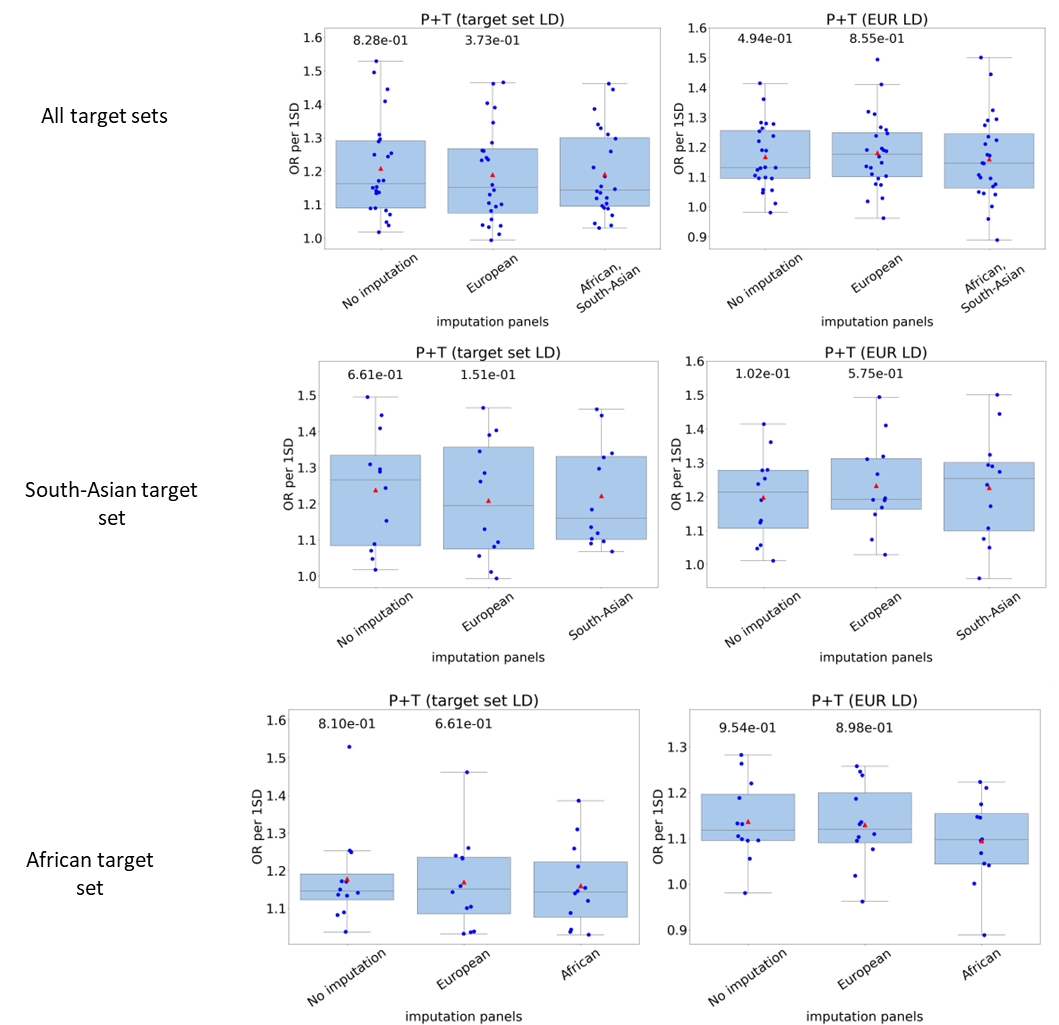
**

**Figure S1.** The effect of ethnic composition of the imputation panels on the performance of P+T (EUR LD) and P+T (target set LD) PRS methods when applied to a target set of a different ethnicity than the GWAS. The graphs show OR per 1SD of 12 traits. PRSs were built from GWASs computed on UKB EUR individuals. Results are shown for both SAS and AFR, SAS only, and AFR only. The p-values above each boxplot compare the results with the imputation panel listed below it to the results with the imputation panel of the target population. P-values were calculated using one-tailed Wilcoxon test. Red triangles are the averages.


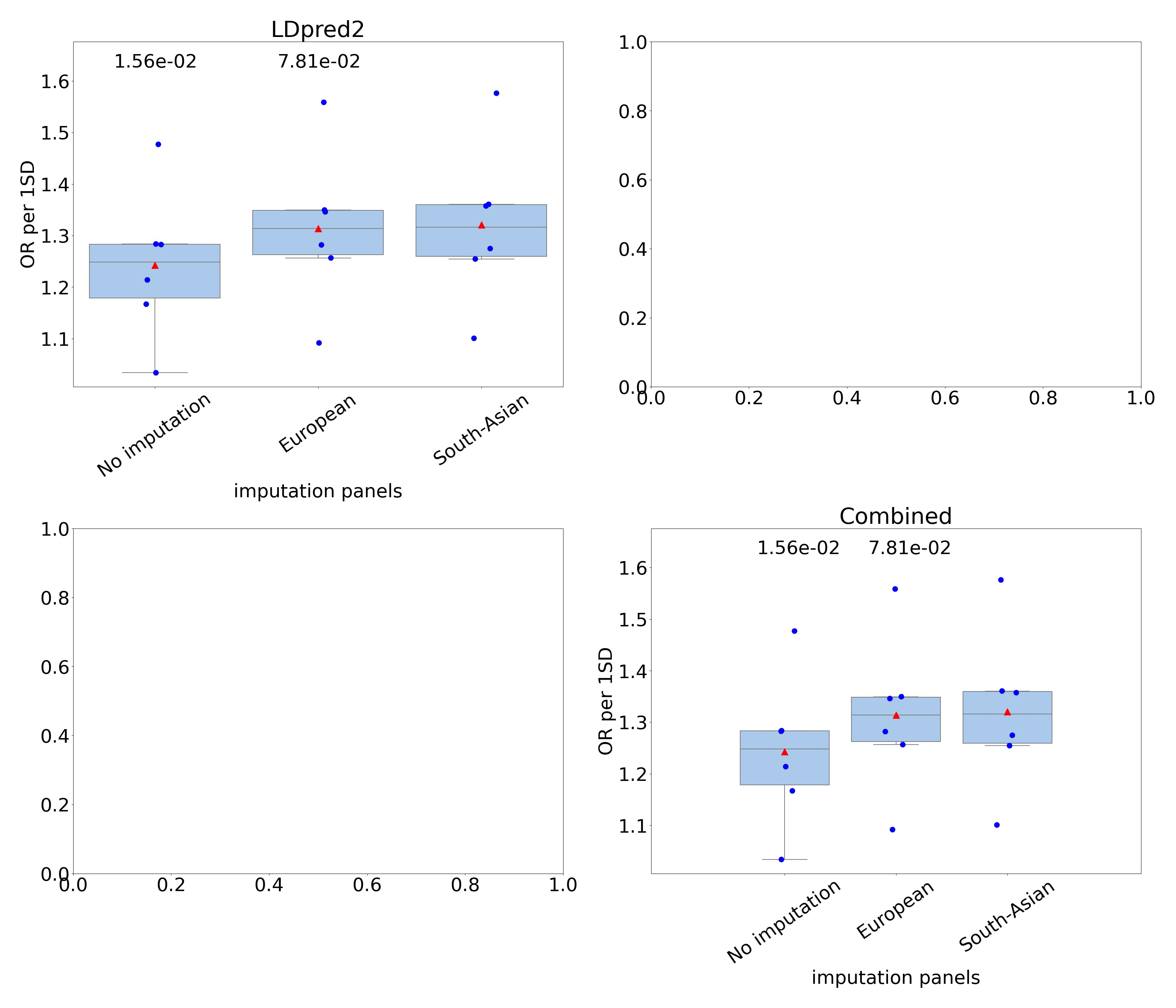


**Figure S2.** The effect of ethnic composition of the imputation panels on the performance of LDpred2 PRS method when applied to SAS target set. PRSs were built from GWASs computed on UKB EUR individuals. The graphs show OR per 1SD of 6 traits. The p-values above each boxplot compare the results with the imputation panel listed below it to the results with the imputation panel of the target population. P-values were calculated using one-tailed Wilcoxon test. Red triangles are the averages.

**
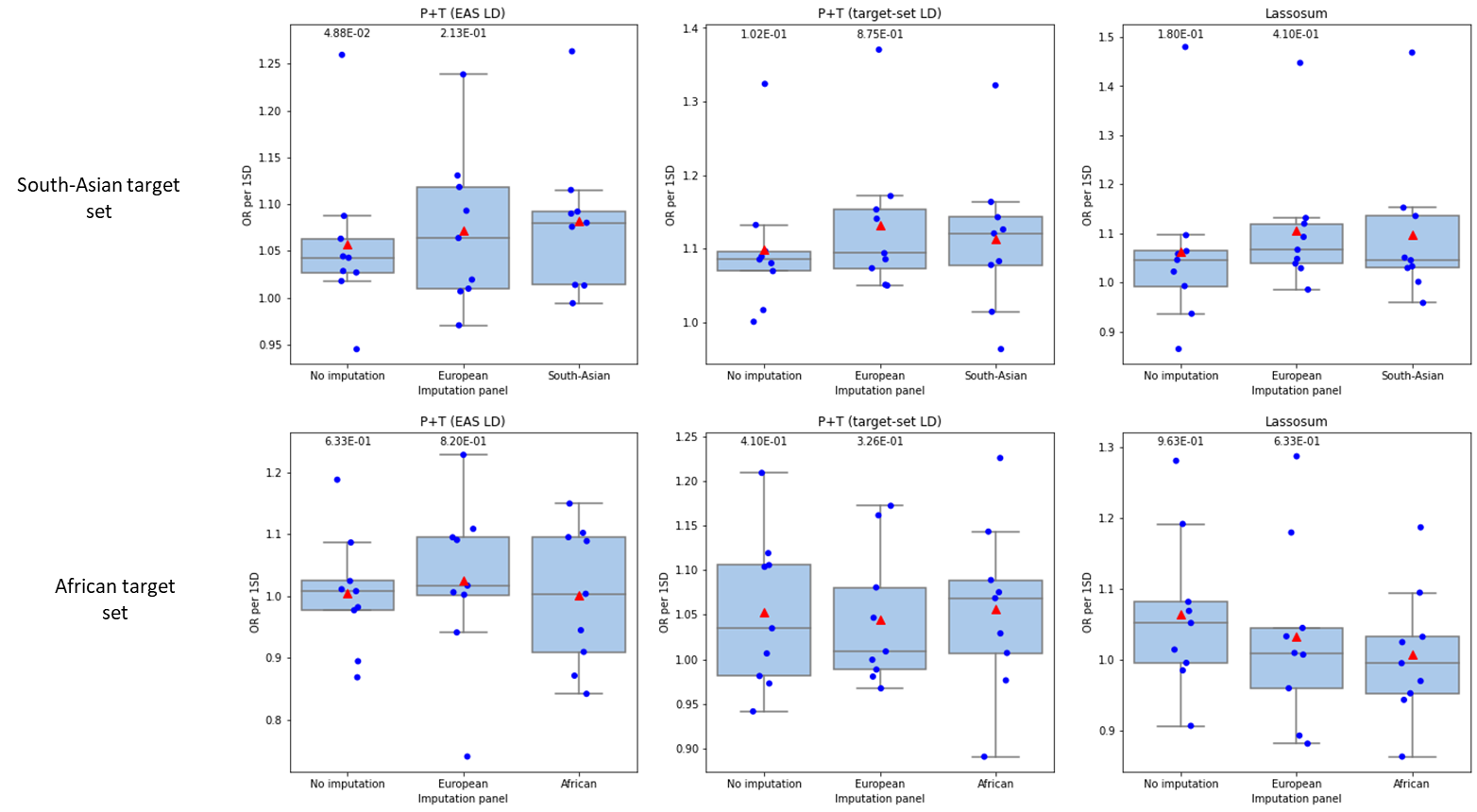
**

**Figure S3.** The effect of the ethnic composition of the imputation panels on PRS performance when applied to EAS-GWASs. Results are shown for PRS methods P+T (EUR LD), P+T (target set LD), and Lassosum. The graphs show OR per 1SD of nine traits. Results are shown for SAS and AFR target sets. The p-values above each boxplot compare the results with the imputation panel listed below it to the results with the imputation panel of the target population. P-values were calculated using one-tailed Wilcoxon test. Red triangles are the averages.


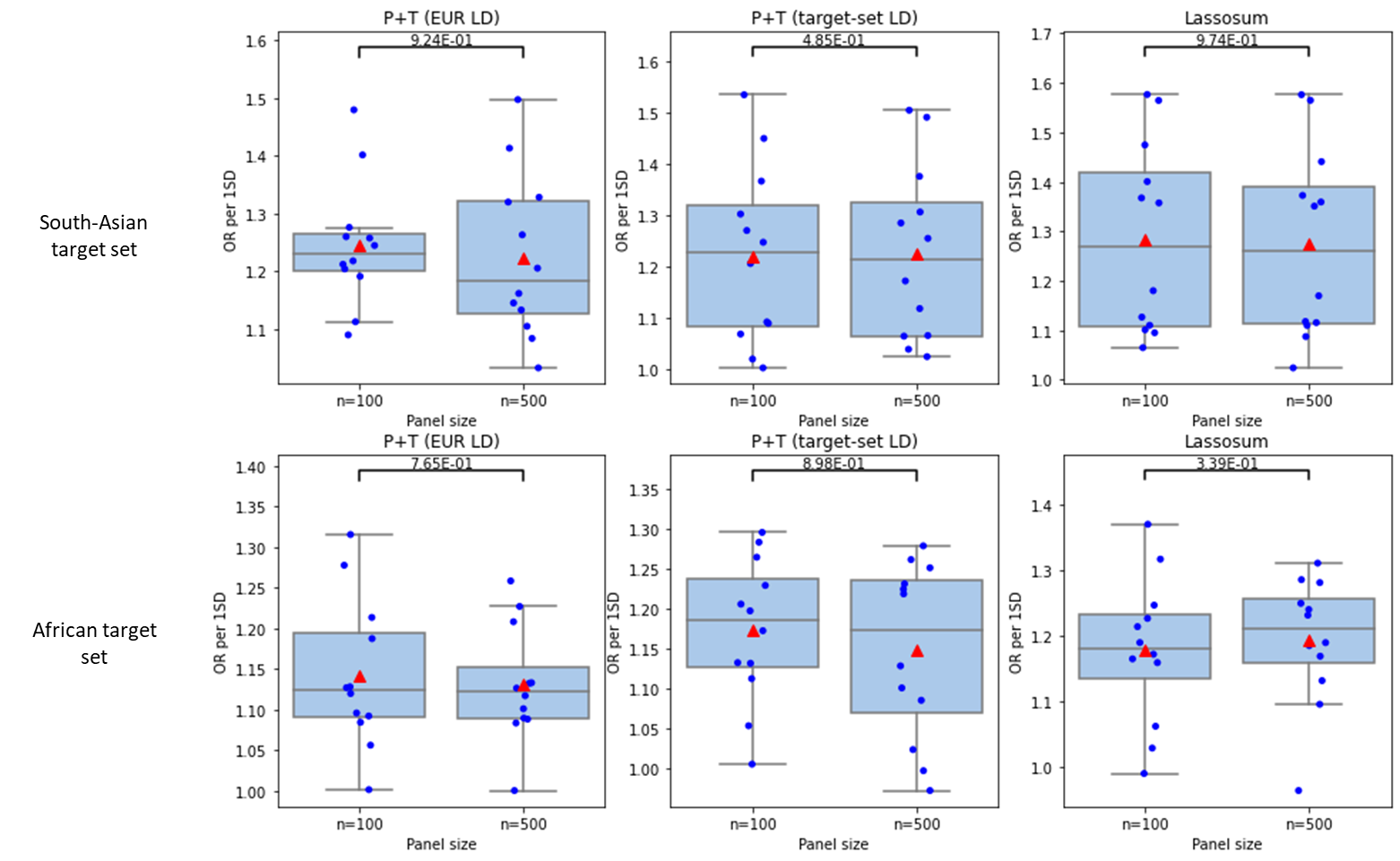


**Figure S4.** The effect of imputation panel size on PRS performance, for PRS methods P+T (EUR LD), P+T (target set LD), and Lassosum. EUR imputation panel of two sizes were tested: n=100 and n=500. The graphs show OR per 1SD of 12 traits. PRSs were built from GWASs computed on UKB EUR individuals. Results are shown for SAS and AFR. The p-values above the boxplots, calculated using one-tailed Wilcoxon test, compare the PRS performance for n=500 and for n=100. Red triangles are the averages.
